# Use of machine-learning algorithms to aid in the early detection of leptospirosis in dogs

**DOI:** 10.1177/10406387221096781

**Published:** 2022-05-21

**Authors:** Krystle L. Reagan, Shaofeng Deng, Junda Sheng, Jamie Sebastian, Zhe Wang, Sara N. Huebner, Louise A. Wenke, Sarah R. Michalak, Thomas Strohmer, Jane E. Sykes

**Affiliations:** Department of Medicine and Epidemiology, University of California–Davis, Davis, CA, USA; School of Veterinary Medicine, and Department of Mathematics, University of California–Davis, Davis, CA, USA; School of Veterinary Medicine, and Department of Mathematics, University of California–Davis, Davis, CA, USA; William R. Pritchard Veterinary Medical Teaching Hospital, University of California–Davis, Davis, CA, USA; William R. Pritchard Veterinary Medical Teaching Hospital, University of California–Davis, Davis, CA, USA; William R. Pritchard Veterinary Medical Teaching Hospital, University of California–Davis, Davis, CA, USA; William R. Pritchard Veterinary Medical Teaching Hospital, University of California–Davis, Davis, CA, USA; William R. Pritchard Veterinary Medical Teaching Hospital, University of California–Davis, Davis, CA, USA; School of Veterinary Medicine, and Department of Mathematics, University of California–Davis, Davis, CA, USA; Department of Medicine and Epidemiology, University of California–Davis, Davis, CA, USA

**Keywords:** artificial intelligence, dogs, infection, kidney, *Leptospira*

## Abstract

Leptospirosis is a life-threatening, zoonotic disease with various clinical presentations, including renal injury, hepatic injury, pancreatitis, and pulmonary hemorrhage. With prompt recognition of the disease and treatment, 90% of infected dogs have a positive outcome. Therefore, rapid, early diagnosis of leptospirosis is crucial. Testing for *Leptospira*-specific serum antibodies using the microscopic agglutination test (MAT) lacks sensitivity early in the disease process, and diagnosis can take >2 wk because of the need to demonstrate a rise in titer. We applied machine-learning algorithms to clinical variables from the first day of hospitalization to create machine-learning prediction models (MLMs). The models incorporated patient signalment, clinicopathologic data (CBC, serum chemistry profile, and urinalysis = blood work [BW] model), with or without a MAT titer obtained at patient intake (=BW + MAT model). The models were trained with data from 91 dogs with confirmed leptospirosis and 322 dogs without leptospirosis. Once trained, the models were tested with a cohort of dogs not included in the model training (9 leptospirosis-positive and 44 leptospirosis-negative dogs), and performance was assessed. Both models predicted leptospirosis in the test set with 100% sensitivity (95% CI: 70.1–100%). Specificity was 90.9% (95% CI: 78.8–96.4%) and 93.2% (95% CI: 81.8–97.7%) for the BW and BW + MAT models, respectively. Our MLMs outperformed traditional acute serologic screening and can provide accurate early screening for the probable diagnosis of leptospirosis in dogs.

Leptospirosis is a zoonotic bacterial disease found worldwide that continues to be associated with sporadic outbreaks in pet dogs in the United States.^14,29^ Over 64 genospecies of motile spirochetes in the genus *Leptospira* have been recognized as causative agents of leptospirosis, with serovars of the species *Leptospira interrogans* and *Leptospira kirschneri* most commonly causing disease in dogs.^7,31^ The bacteria are maintained in the renal tubules of reservoir hosts, especially rodents, and are shed intermittently in the urine.^2,33^ Large-scale epidemiologic studies are lacking for canine leptospirosis, hence prevalence is unknown. However, one study determined that 5.4% of specimens submitted to a commercial laboratory for PCR testing were positive for *Leptospira* DNA.^27^ Further, up to 20% of dogs in some populations have detectable *Leptospira* antibodies,^9,22,30^ and 1–13% of healthy dogs in developed countries are shedding leptospires in their urine.^3,10,18,24,28^

Clinical manifestations of leptospirosis can include fever, variable acute hepatic injury and renal failure, pancreatitis, and hemorrhagic pulmonary disorders.^31^ These clinical signs, with concurrent hematologic and biochemical findings consistent with leptospirosis, may raise the index of suspicion for leptospirosis, but a diagnosis is often made retrospectively given the pitfalls of the available detection tools. Several commercial laboratories offer *Leptospira* PCR assays that can be performed on blood or urine specimens; however, false-negative results can occur depending on the phase of disease or previous administration of antimicrobials; therefore, a negative PCR result alone should not be used to rule out the disease.^6,13^ Limited data are available regarding the clinical performance of commercial PCR assays. In one study, blood PCR exhibited a sensitivity of 86% up to 6 d post-infection, but decreased to 34% after 1 wk of infection.^23^ Specificity is thought to be high because assays are designed to detect gene segments that are specific to pathogenic *Leptospira* species.^6,13^ Paired acute- and convalescent-phase serology using the microscopic agglutination test (MAT) is the serologic reference standard for diagnosis of leptospirosis, with a sensitivity of 100%. However, negative MAT titers are common during the first week of infection, and the sensitivity of a single acute MAT titer is only 50%.^8,21^ A single positive MAT titer can indicate previous exposure or recent vaccination; thus, unless very high (>1:3,200), a single positive MAT titer lacks specificity.^19^

There is a critical need for improved sensitivity of early detection of leptospirosis, given that dogs with leptospirosis may need intensive care or referral services, including hemodialysis. The prognosis for dogs with leptospirosis is good.^1^ In contrast, the prognosis for other disease processes resulting in severe kidney injury may be poorer.^25,26^ In cases that require intensive care, having a rapid diagnosis and accurate prognostic information are critical in the decision-making process of clients to pursue advanced therapies. A retrospective diagnosis of leptospirosis may delay appropriate therapy, increase cost to the owner, and potentially result in early euthanasia despite the presence of a treatable disease. We have observed common patterns of abnormalities on bloodwork and urinalysis in affected dogs that raises suspicion for leptospirosis. We aimed to utilize MLMs to develop a novel leptospirosis prediction model based on patient signalment and clinicopathologic findings available on the first day of hospitalization in dogs with a clinical suspicion to improve the early diagnosis of leptospirosis in dogs.

## Materials and methods

### Patient selection

We searched the electronic medical record system of the University of California–Davis Veterinary Medical Teaching Hospital (VMTH) for all dogs with ≥1 *Leptospira* MAT performed between January 1, 2000 and December 31, 2020. We included dogs if a CBC (Advia 120; Siemens), serum chemistry panel (Cobas c501/6000; Roche), and urinalysis (Urine Chemstrip, Urisys 1800, Cobas U411; Roche) were performed during the initial patient encounter in which a MAT was submitted. The VMTH Clinical Laboratory Services conducted all clinicopathologic testing, and MATs were performed at the adjacent California Animal Health and Food Safety Laboratory, using *Leptospira interrogans* serovars Bratislava, Canicola, Pomona, Icterohaemorrhagiae, and Hardjo, and *Leptospira kirschneri* serovar Grippotyphosa, as antigens. The complete medical record was reviewed for all patients, and patient data were extracted, including signalment and recent vaccination status. Dogs were excluded from analysis if their weight, sex, or breed was not included in the medical record. Institutional animal care and use approval was not needed for this retrospective analysis of clinical data.

**Figure 1.**
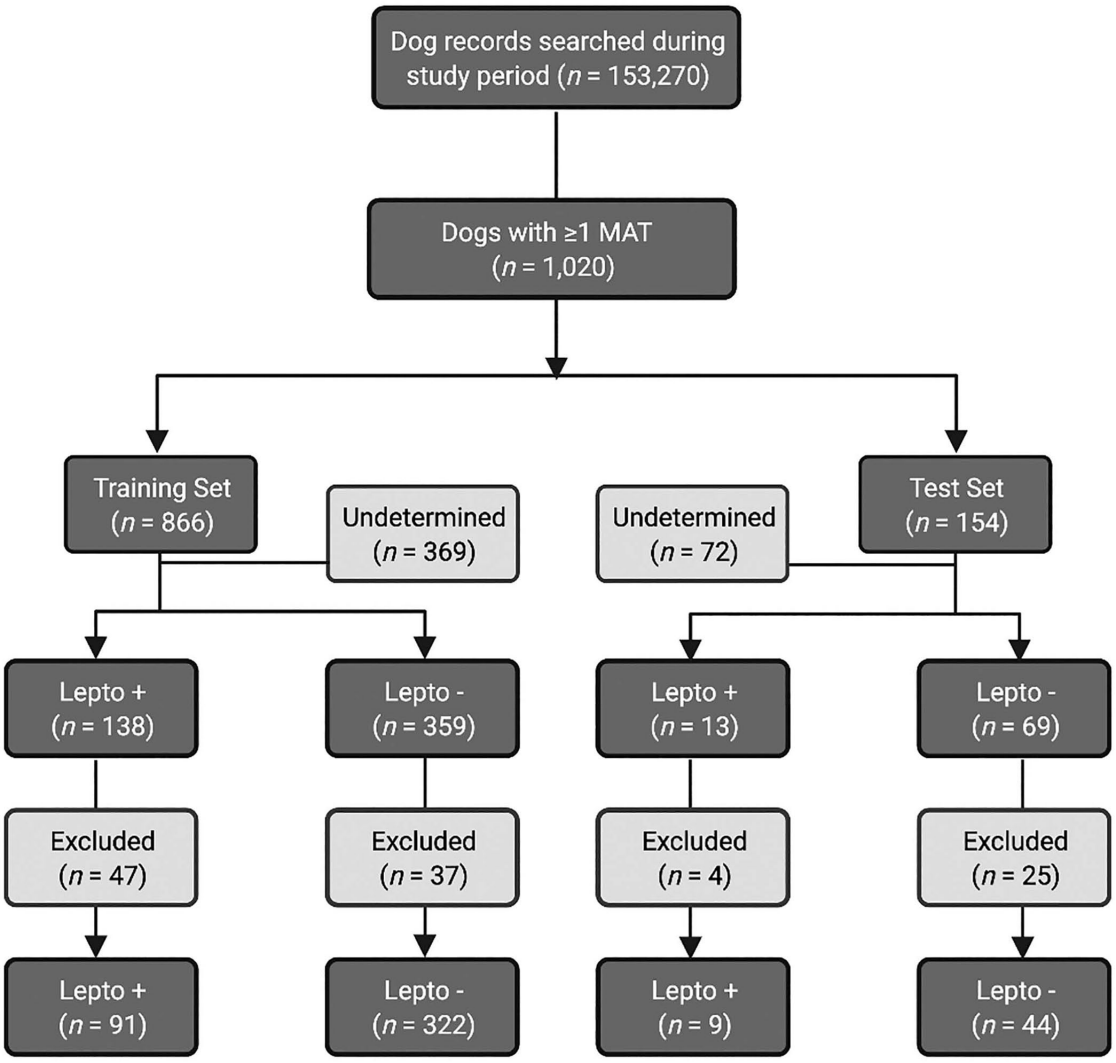
Consort diagram illustrating patient enrollment, categorization of leptospirosis classification, and inclusion into training or test set data. Lepto = leptospirosis.

The model was trained and validated on data collected from all dogs seen between January 1, 2000 and December 31, 2018 (training set). The algorithm was then tested on data collected from dogs seen between January 1, 2019 and December 31, 2020 (test set; Fig. 1).

Normality testing was performed for clinicopathologic data from dogs in the training and test sets. Descriptive, nonparametric statistics of the data were performed to compare clinical features between positive and negative cases. Continuous features were compared with a Mann–Whitney test and categorical features with a Fisher exact test. The *p*-values for multiple comparisons of features were corrected using the Bonferroni–Dunn method, and the adjusted *p*-values are reported. An adjusted *p* ≤ 0.05 is considered significant (Prism v.9.2.0; GraphPad).

### Reference standard leptospirosis classification

Dogs were classified as leptospirosis-positive, -negative, or -undetermined based on specific *Leptospira* testing. Dogs were considered leptospirosis-positive if there were compatible clinical features such as evidence of renal injury, hepatic injury, vasculitis, or uveitis, and 1) a ≥4-fold increase in MAT titer to any serovar between acute and convalescent specimens, 2) a single MAT ≥1:3,200 in the absence of reported vaccination, or 3) positive blood or urine *Leptospira* PCR tests. Dogs were considered leptospirosis-negative if 1) a <4-fold increase was observed between acute and convalescent MAT titers to any one serovar, 2) a single convalescent MAT titer was ≤1:100 in the absence of vaccination, or 3) convalescent MAT titers were ≤1:400 in a vaccinated dog. Dogs that did not meet either of these sets of criteria were considered leptospirosis-undetermined and excluded from further analysis.

### Feature preprocessing

Patient data, including breed, sex, weight, and clinicopathologic parameters, were utilized as features (Table 1). Numerical, continuous features were all standardized, subtracting the mean and dividing by the SD of the feature. Sex was encoded using one-hot encoding (0 and 1) then standardized as above. One-hot encoding of dog breeds yielded an excessive number of dimensions; therefore, dog breeds were grouped into 1 of 10 groups based on AKC breed group determination: toy, herding, hound, non-sporting, sporting, terrier, working, foundation stock service, mix breed, or other. The prior probability for each group was determined as the leptospirosis-positive rate of the group in the training set. For groups with fewer than 5 members, the prior probability for the group was the leptospirosis-positive rate of the entire training set. The feature was then normalized by the SD.

**Table 1.** Signalment and clinicopathologic features utilized for training of the machine-learning models.

Demographic	CBC	Serum chemistry	Urinalysis
Breed group	Hematocrit	Anion gap	Urine specific gravity
Weight (kg)	Hemoglobin	Sodium	Urine protein (0–4+)
Sex	MCV	Potassium	Urine glucose (0–4+)
	White blood cells	Chloride	
	Band neutrophils	Bicarbonate	
	Neutrophils	Phosphorus	
	Lymphocytes	Calcium	
	Monocytes	Urea	
	Eosinophils	Creatinine	
	Platelets	Glucose	
		Total protein	
		Albumin	
		Globulin	
		ALT	
		AST	
		ALP	
		GGT	
		Cholesterol	
		Bilirubin	

ALP = alkaline phosphatase; ALT = alanine transaminase; AST = aspartate transaminase; GGT = gamma-glutamyl transferase; MCV = mean corpuscular volume.

### Machine-learning model training

Two leptospirosis prediction models were trained. The blood work (BW) model was trained with all patient features and clinicopathologic parameters (Table 1). The second model, BW + MAT, incorporated the initial hospitalization MAT titer in addition to the patient features and clinicopathologic parameters (Table 1).

**Figure 2.**
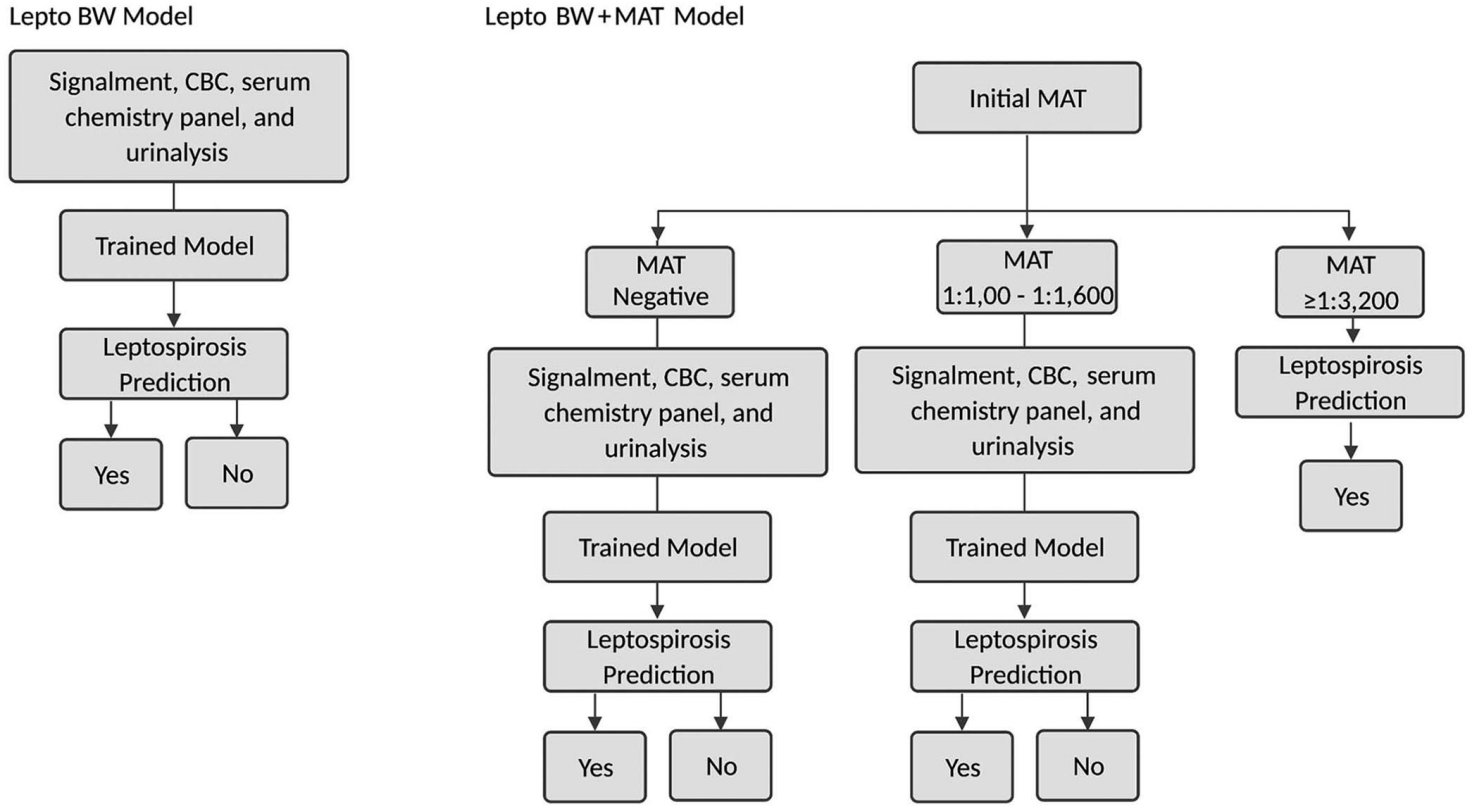
Overview of workflow for the 2 prediction models, blood work (BW) and BW + microscopic agglutination test (MAT).

The BW model (Fig. 2) utilized a single machine-learning algorithm. Using the training set data, a support vector machine (SVM) algorithm with radial basis function kernel was implemented (v.0.24.1, Python3; scikit-learn).^5^ The model was trained using a repeated 10-fold cross-validation process repeated 100 times to produce the average confusion matrix to tune the hyperparameters. Hyperparameters were tuned using a linear grid search and optimized based on the G-mean, the geometric mean of sensitivity and specificity. The optimal hyperparameters were γ (kernel coefficient) = 0.028, C (regularization parameter) = 0.26, and r (positive-to-negative class weight) = 5.4.

The BW+MAT model first utilizes a static decision tree to stratify patients based on the MAT titer obtained at first hospitalization (Fig. 2). Once patients were stratified based on MAT titer, a prediction model was trained. For dogs with a negative MAT, a nonlinear SVM model was trained as for the BW model. For dogs with a MAT of 1:100–1:1,600, a nonlinear SVM model was trained using signalment and clinicopathologic parameters (Table 1) and the initial MAT titer. The MAT titer was log-transformed and normalized to the SD. Ten-fold cross-validation was performed 100 times, and optimal hyperparameters were determined using a linear grid search (γ = 0.030, C = 0.10, r = 7.8). A prediction of leptospirosis-positive was made for all dogs with a MAT ≥1:3,200. The code utilized to build this model is publicly available (https://github.com/sf-deng/lepto-classifier).

### Machine-learning model performance evaluation

The test set included dogs that were evaluated in the 2 y after the SVM model was trained and served as a prospective evaluation of the model. The hyperparameters optimized on the training set were utilized during BW and BW+MAT model testing. Model prediction results are reported as sensitivity and specificity. Predictions made were utilized as a binary classifier, a receiver operating characteristic (ROC) plot was generated, and area under the curve (AUC) was calculated. The 95% CIs of the sensitivity, specificity, and AUC were calculated using the Wilson–Brown method (Prism v.9.2.0; GraphPad).

## Results

### Study population demographics

During the study period, 15,3270 unique dogs were evaluated at the VMTH. Of these dogs, 1,020 were tested for leptospirosis: 866 in the training set, and 154 in the test set. In the training set, 138 dogs were classified as leptospirosis-positive, 359 were leptospirosis-negative, and 369 were leptospirosis-undetermined. Dogs were excluded if complete blood work or demographic data was not available, leaving 91 leptospirosis-positive dogs and 322 leptospirosis-negative dogs in the training set. Thirteen dogs were leptospirosis-positive in the test set, 69 were leptospirosis-negative, and 72 were leptospirosis-undetermined. After excluding dogs with incomplete demographic data or blood work, the test set contained 9 leptospirosis-positive dogs and 44 leptospirosis-negative dogs (Fig. 1).

In the leptospirosis-positive population, the median highest *Leptospira* MAT titer obtained at the time of first evaluation was 1:1,600 (range: negative to 1:102,400) in the training set and 1:3,200 (range: 1:800–1:3,200) in the test set (Table 2). In leptospirosis-positive dogs, 57 dogs in the training set and 1 dog in the test set had acute MAT titers <1:3,200. In the leptospirosis-negative groups, the median highest *Leptospira* MAT titer at the time of first evaluation was 1:100 (range: negative to 1:1,600) in the training set and 1:100 (range: negative to 1:3,200) in the test set. An initial MAT of ≥1:3,200 was 42% sensitive (95% CI: 32.8–51.8%), with an AUC of 0.775 (95% CI: 0.700–0.850) for the prediction of leptospirosis in the entire study population. Specificity was not calculated because an initial MAT of ≥1:3,200 was used as a component of the gold standard for diagnosis of leptospirosis.

**Table 2.** The method of leptospirosis diagnosis or exclusion for dogs in the training and test sets.

Methodology	Training set, *n* (%)		Test set, *n* (%)	
	Positive	Negative	Positive	Negative
Positive diagnosis				
4-fold increase between acute and convalescent MAT	57 (62.6)		3 (33.3)	
Single MAT ≥1:3,200 in the absence of vaccination	24 (26.4)		3 (33.3)	
4-fold increase between acute and convalescent MAT, and positive PCR	7 (7.7)		0 (0)	
Single MAT ≥1:3,200 in the absence of vaccination, and positive PCR	3 (3.3)		3 (33.3)	
Negative diagnosis				
<4-fold increase between acute and convalescent MAT		125 (38.8)		14 (31.8)
Single convalescent MAT ≤1:100		164 (50.9)		26 (59.1)
Vaccinated with a single convalescent MAT ≤1:400		33 (10.2)		4 (9.1)

Of the leptospirosis-positive dogs in the training set, 57 of 91 (63%) were male, with a median age of 7.5 y (range: <1–16 y) and weight of 24 kg (range: 3–57 kg; Table 3). Mixed-breed dogs represented 26 of 91 (29%) dogs with leptospirosis; working and sporting group dogs were the purebred dogs observed most frequently with leptospirosis. In the leptospirosis-negative training set, 139 of 322 (43%) were male, with a median age of 7 y (range: <1–16 y) and weight of 22.4 kg (range: 2–86 kg). Sporting dogs represented the highest proportion of leptospirosis-negative dogs, followed by mixed-breed dogs.

**Table 3.** Summary of patient demographics of dogs in the training and test sets.

Characteristics	Training set, *n* (%)		Test set, *n* (%)	
	Positive	Negative	Positive	Negative
Sex				
Female	34 (37)	183 (57)	4 (44)	26 (59)
Male	57 (63)	139 (43)	5 (56)	18 (40)
Breed group				
Foundation stock service	1 (1)	0 (0)	0 (0)	0 (0)
Herding	13 (14)	34 (11)	2 (22)	3 (7)
Hound	8 (9)	13 (4)	1 (11)	0 (0)
Mix	26 (29)	70 (22)	3 (33)	13 (30)
Non-sporting	2 (2)	35 (11)	0 (0)	3 (7)
Other	1 (1)	2 (1)	0 (0)	0 (0)
Sporting	12 (13)	89 (28)	1 (11)	11 (25)
Terrier	7 (8)	31 (10)	2 (22)	2 (5)
Toy	6 (7)	26 (8)	0 (0)	7 (16)
Working	15 (16)	22 (7)	0 (0)	5 (11)
Age category (y)				
<3	16 (18)	55 (18)	1 (11)	3 (7)
3 to <6	20 (22)	63 (20)	2 (22)	8 (18)
6 to <9	22 (24)	88 (27)	4 (44)	11 (25)
9 to <12	25 (27)	65 (21)	2 (22)	9 (21)
≥12	8 (9)	51 (16)	0 (0)	13 (30)
Weight category (kg)				
<10	17 (19)	84 (26)	2 (22)	13 (30)
10 to <20	16 (18)	61 (19)	1 (11)	6 (14)
20 to <30	22 (24)	83 (26)	2 (22)	11 (25)
30 to <40	24 (26)	68 (21)	2 (22)	10 (23)
≥40	12 (13)	26 (8)	2 (22)	4 (9)

In the test set, 5 of 9 (56%) of the leptospirosis-positive dogs were male (Table 3). The median age was 6 y (range: 2–9 y), and weight was 25.6 kg (range: 4–50 kg). In the leptospirosis-negative test set, 18 of 44 (40%) were male, with a median age of 8.5 y (range: <1–17 y) and weight of 22.5 kg (range: 2–50 kg). Across the entire test set, dogs that were leptospirosis-positive were more likely to be males (*p* = 0.001) and weighed more (*p* = 0.03) than dogs that were leptospirosis-negative. There was no significant difference in age between the 2 groups (*p* = 0.4).

### Clinicopathologic findings

In the leptospirosis-positive dogs included in the training set, anemia (Hct <0.4 L/L) was noted in 69 of 91 (76%) dogs, microcytosis (MCV <65 fL) was noted in 17 of 91 (19%) dogs, and neutrophilia (>11 × 10^9^/L) was detected in 52 of 91 (57%) dogs. In the leptospirosis-negative group, anemia was noted in 213 of 322 (66%) dogs, microcytosis was noted in 44 of 322 (14%) dogs, and neutrophilia was detected in 111 of 322 (34%) dogs. Leptospirosis-positive dogs had lower MCVs (*p* < 0.05), higher total leukocyte counts (*p* < 0.05), and higher neutrophil counts (*p* < 0.05; Table 4) than leptospirosis-negative dogs. In the leptospirosis-positive test set, anemia was noted in 4 of 9 (44%) dogs, microcytosis in 1 of 9 (11%) dogs, and neutrophilia in 4 of 9 (44%) dogs. In the leptospirosis-negative test set, anemia was noted in 8 of 44 (18%) dogs, microcytosis in 2 of 44 (4 %) dogs, and neutrophilia in 4 of 44 (9%) dogs. Across the entire test set, anemia and neutrophilia were more likely to be detected in leptospirosis-positive dogs (*p* = 0.026 and *p* < 0.001, respectively) than in leptospirosis-negative dogs.

**Table 4.** Clinicopathologic data for dogs with and without leptospirosis in the training and test sets used to train machine-learning models.

Characteristic	Training set median			Test set median		
	Positive	Negative	Adjusted *p* value	Positive	Negative	Adjusted *p* value
CBC						
Hematocrit (L/L)	0.29 (0.18–0.34)	0.36 (0.29–0.42)	1	0.40 (0.30–0.47)	0.38 (0.33–0.43)	1
Hemoglobin (g/L)	100 (64–120)	120 (100–140)	1	130 (100–160)	130 (110–140)	1
MCV (fL)	66 (58–67)	69 (67–72)	0.003	67 (65–69)	70 (68–73)	0.227
Leukocytes (×10^9^/L)	12 (8–15)	11 (8.5–17)	0.003	14 (11–17)	11 (7.8–15)	1
Band neutrophils (×10^9^/L)	0 (0–0)	0 (0–0)	1	0 (0–0)	0 (0–0)	1
Neutrophils (×10^9^/L)	9.1 (6.1–11)	8.6 (5.9–13)	0.003	11 (8.8–14)	8.8 (6.2–13)	1
Lymphocytes (×10^9^/L)	1.1 (0.2–1.6)	1.3 (0.8–1.7)	0.074	1.4 (1.1–1.7)	1.1 (0.5–1.8)	1
Monocytes (×10^9^/L)	0.6 (0.2–0.9)	0.6 (0.4–1.1)	0.003	936 (557–1,446)	0.7 (0.4–1.0)	1
Eosinophils (×10^9^/L)	0 (0–0.1)	0.2 (0.1–0.4)	0.048	0 (0–0.3)	0.2 (0.1–0.4)	1
Platelets (×10^9^/L)	126 (0–179)	247 (143–363)	0.272	210 (132–319)	295 (159–418)	1
Serum chemistry panel						
Anion gap (mmol/L)	25 (13–30)	27 (22–31)	0.026	31 (27–35)	25 (20–31)	0.854
Sodium (mmol/L)	141 (131–144)	147 (144–150)	0.003	143 (143–146)	147 (146–151)	0.026
Potassium (mmol/L)	3.7 (2.9–4.2)	4.6 (4–5.1)	0.531	4.1 (3.7–4.7)	4.6 (4.2–5.1)	1
Chloride (mmol/L)	97 (79–102)	109 (104–112)	0.003	97 (96–103)	109 (104–113)	0.003
Bicarbonate (mmol/L)	15 (5–17)	17 (14–21)	1	18 (14–22)	17 (14–21)	1
Phosphorus (mmol/L)	2.6 (1.1–3.9)	2.9 (1.8–4.8)	0.189	4.2 (1.9–5.2)	2.2 (1.5–3.6)	1
Calcium (mmol/L)	2.4 (1.8–2.5)	2.8 (2.4–3)	0.586	2.5 (2.5–2.8)	2.8 (2.5–3.0)	1
Urea (mmol/L)	34 (4.6–50)	38 (19–56)	0.003	44 (32–72)	25 (11–47)	0.864
Creatinine (µmol/L)	433 (80–636)	486 (256–822)	0.08	592 (248–1237)	327 (159–530)	1
Glucose (mmol/L)	5.1 (3.4–5.8)	5.6 (5.0–6.2)	1	5.9 (5.4–6.7)	5.8 (5.1–6.5)	1
Total protein (g/L)	49 (39–54)	57 (48–64)	1	66 (57–74)	58 (51–63)	1
Albumin (g/L)	24 (19–26)	28 (24–34)	0.778	32 (27–35)	33 (29–37)	1
Globulin (g/L)	23 (17–28)	27 (23–32)	1	34 (25–40)	25 (22–28)	0.224
ALT (U/L)	46 (3–81)	64 (34–164)	1	69 (50–167)	107 (42–265)	1
AST (U/L)	54 (25–87)	52 (32–95)	0.003	72 (48–172)	52 (31–93)	1
ALP (U/L)	66 (18–110)	113 (47–307)	1	142 (109–457)	138 (44–379)	1
GGT (U/L)	3 (0–4)	3 (3–8)	1	4 (0–7)	3 (0–8.8)	1
Cholesterol (mmol/L)	4.6 (2.7–5.9)	6.4 (4.9–8.0)	1	5.7 (5.1–7.1)	6.7 (4.8–7.7)	1
Bilirubin (µmol/L)	3.4 (1.7–5.1)	3.4 (1.7–6.8)	0.003	3.4 (3.4–5.1)	3.4 (3.4–3.4)	1
Urinalysis						
Urine specific gravity	1.012 (1.010–1.015)	1.012 (1.010–1.015)	1	1.012 (1.011–1.014)	1.013 (1.010–1.017)	1
Urine protein (0–4)	1 (0–2)	1 (1–3)	1	2 (1–3)	1 (1–2)	1
Urine glucose (0–4)	0 (0–1)	0 (0–0)	0.003	2 (0–2)	0 (0–0)	0.125

Numbers in parentheses are interquartile ranges. *p*-values adjusted with Bonferroni–Dunn method.

The serum chemistry panel results revealed an increased creatinine concentration (>124 µmol/L) in 89 of 91 (98%) and hyperbilirubinemia (>3.4 µmol/L) in 52 of 91 (57%) of the leptospirosis-positive dogs in the training set. For the leptospirosis-negative dogs in the training set, an increased creatinine concentration was noted in 278 of 322 (86%) dogs and hyperbilirubinemia in 116 of 322 (36%) dogs. In the leptospirosis-positive test set, an increased creatinine concentration was noted 9 of 9 (100%) dogs, and hyperbilirubinemia in 4 of 9 (44%). In the leptospirosis-negative dogs in the test set, an increased creatinine concentration was noted in 37 of 44 (84%) dogs and hyperbilirubinemia in 9 of 44 (20%) dogs. In the entire population, azotemia and hyperbilirubinemia were more commonly observed in leptospirosis-positive dogs than leptospirosis-negative dogs (both *p* < 0.001). In both the training and test sets, sodium (adjusted *p* = 0.003 and adjusted *p* = 0.026) and chloride (both adjusted *p* = 0.003) concentrations were significantly lower in leptospirosis-positive dogs compared to leptospirosis-negative dogs (Table 4).

Urinalysis revealed proteinuria in 80 of 91 (88%) and glucosuria in 65 of 91 (71%) leptospirosis-positive dogs in the training set. Of the leptospirosis-negative dogs in the training set, 254 of 322 (79%) had proteinuria, and 73 of 322 (23%) had glucosuria. Of the leptospirosis-positive dogs in the test set, 9 of 9 (100%) had proteinuria, and 6 of 9 (67%) had glucosuria. Of the leptospirosis-negative dogs in the test set, 32 of 44 (73%) had proteinuria, and 7 of 44 (16%) had glucosuria. Of the entire data set (all dogs in training and test sets), leptospirosis-positive dogs were more likely to have proteinuria and glucosuria than leptospirosis-negative dogs (*p* = 0.02 and adjusted *p* < 0.001, respectively) and leptospirosis-positive dogs within the training set had higher urine glucose concentrations compared to leptospirosis-negative dogs (adjusted *p* = 0.003; Table 4).

### Machine-learning model performance

**Figure 3.**
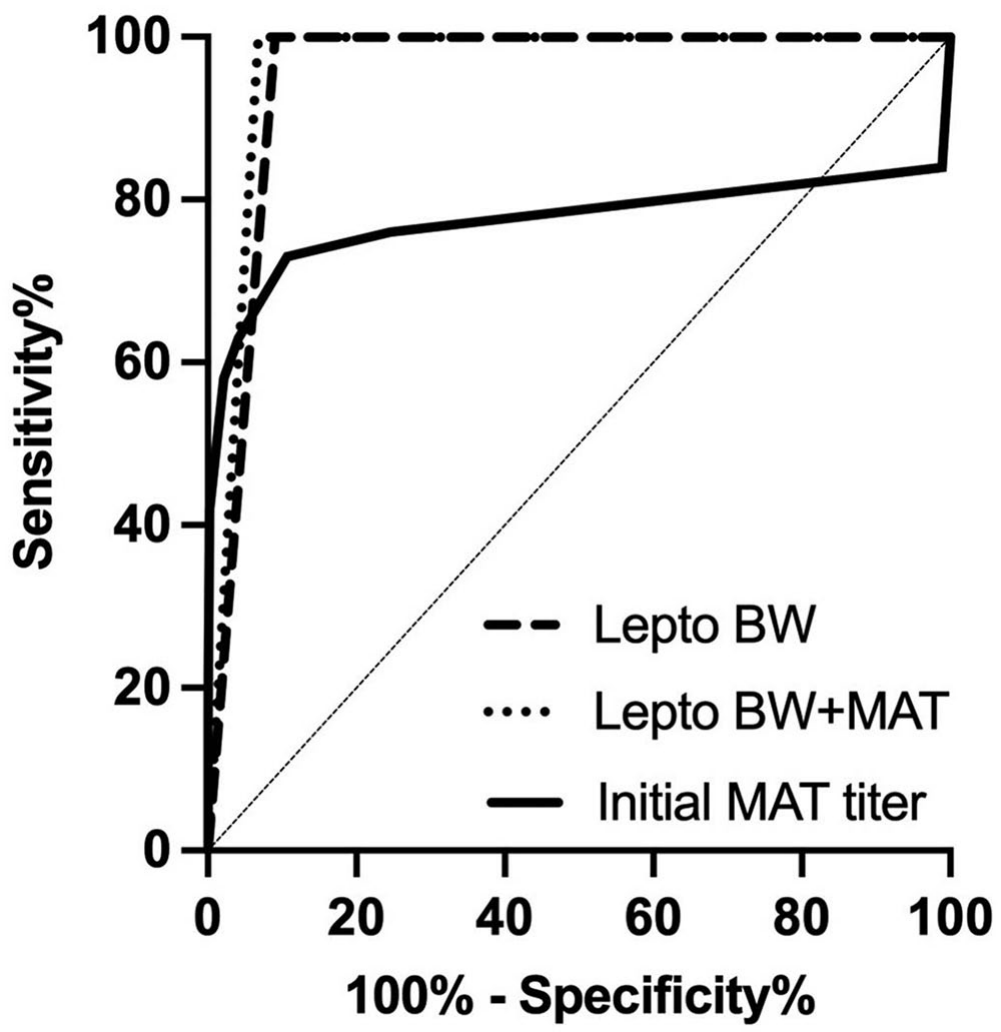
Receiver operator characteristic curves for blood work (BW) and BW + microscopic agglutination test (MAT) performance on the test set data and the MAT titer collected at initial hospitalization.

The BW model accurately predicted a leptospirosis diagnosis in 49 of 53 (92%) dogs in the test set (Table 5). A prediction of leptospirosis was made in all 9 leptospirosis-positive dogs with a sensitivity of 100% (95% CI: 70.1–100%). A negative prediction was made in 40 of 44 of the leptospirosis-negative dogs, resulting in a specificity of 90.9% (95% CI: 78.8–96.4%). The positive and negative likelihood ratios are 11.0 and 0, respectively. The BW model has an AUC of 0.955 (95% CI: 0.901–1.00; Fig. 3).

**Table 5.** The performance of machine-learning models BW and BW+MAT and initial MAT titer on the test set.

Leptospirosis prediction method	Sensitivity (%)	Specificity (%)	AUC
Machine-learning model: BW	100 (70.1–100)	90.9 (78.8–96.4)	0.955 (0.901–1.00)
Machine-learning model: BW + MAT	100 (70.1–100)	93.2 (81.8–97.7)	0.959 (0.920–1.00)
Initial MAT ≥1:3,200	42 (32.8–51.8)	NA	0.775 (0.700–0.850)

NA = not applicable. Numbers in parentheses are 95% CIs.

The BW+MAT model accurately predicted leptospirosis in 9 of 9 leptospirosis-positive dogs and had a sensitivity of 100% (95% CI: 70.1–100%). The BW + MAT model predicted a negative status in 41 of 44 leptospirosis-negative dogs and had a specificity of 93.2% (95% CI: 81.8–97.7%). The positive and negative likelihood ratios are 14.7 and 0, respectively. The trained BW + MAT model had an AUC of 0.959 (95% CI: 0.920–1.00; Table 5).

## Discussion

Using patient information and clinicopathologic data available on the first day of hospitalization, our MLMs predicted a diagnosis of leptospirosis in dogs with a clinical suspicion of the disease with high accuracy. Incorporation of the first available MAT titer in this MLM did not improve the predictive power in a clinically relevant manner; patterns present in hematologic and biochemical parameters can differentiate between leptospirosis and other disease processes without *Leptospira-*specific tests. When compared to a single MAT titer, sensitivity was improved from 42% to 100% and AUC from 0.775 to >0.95. MLMs can provide rapid insight into a patient’s diagnosis to guide clinical decision-making.

The index of suspicion for leptospirosis may be increased based on patterns present on abnormalities in routine screening tests (i.e., CBC, serum biochemistry, urinalysis), that are associated with the infection. The most common biochemical finding in dogs with leptospirosis is azotemia, present in 80–100% of cases,^4,12,20,32^ and in nearly all of our cases. Elevations in serum liver enzyme activities are present in 50% of cases, similar to our population, but only rarely in the absence of azotemia.^4,11,12,15,20^ Evidence of muscle injury is noted by an elevation in creatine kinase in 43% of dogs with leptospirosis.^20^ Electrolyte abnormalities, including hyponatremia and hypochloremia, can be observed, but serum potassium levels can paradoxically remain normal in the face of oligoanuria as a result of inhibition of sodium/potassium–ATPase produced by bacterial endotoxins.^15,16,34^ Evidence of inflammation may be present, including hypoalbuminemia, neutrophilia, or lymphopenia.^4,15^ Thrombocytopenia is reported in 20–50% of dogs with leptospirosis.^4,12,20,32^ Common urinalysis findings include urine specific gravity in the hyposthenuric or isosthenuric range, and proteinuria, with or without glucosuria, similar to our leptospirosis-positive population.^4,11,15,32^ The reported hematologic and biochemical parameter changes in our study population were similar to these published results, indicating our population has clinicopathologic changes similar to other populations of dogs with leptospirosis. The MLMs can utilize these routine screening tools to differentiate between leptospirosis and other causes of illness.

Dogs with positive blood or urine *Leptospira* PCR results with concomitant clinical features consistent with leptospirosis were categorized as leptospirosis-positive. PCR was not widely used for diagnosis of leptospirosis in this population, likely because of the broad application of empiric antimicrobials prior to referral and associated lack of sensitivity in that situation. Therefore, comparisons between the sensitivity of PCR and MLMs were not possible.

Our criteria for a diagnosis incorporated results of PCR and MAT titers, clinical features, and reported vaccine history. These stringent criteria resulted in a substantial proportion of dogs with a leptospirosis-undetermined status given lack of completion of confirmatory testing. Published guidelines recommend performance of paired acute and convalescent MAT in dogs suspected of having leptospirosis; however, dogs may be lost to follow-up or clients may decline this testing. Although our criteria were designed to distinguish stringently between leptospirosis-positive and -negative dogs, the retrospective nature of our study and our reliance upon owner-reported history of vaccination may have led to some misclassification of patients.

A limitation of our study is that all dogs were included because they were tested for leptospirosis by the attending clinician. Consequently, these models are only trained to differentiate between leptospirosis and other disease processes that mimic leptospirosis enough to warrant testing. Applying these MLMs to a broader population may impact the positive predictive value by including those with a lower pretest probability of having leptospirosis. In addition, the control population primarily consisted of dogs with renal disease (>85% azotemic), but this likely represents a heterogeneous group of disease processes that were not readily ascertained because of the lack of gold standard tests in all cases. With this limitation, it is not possible to predict an accurate clinical diagnosis in the leptospirosis-negative group, but it does demonstrate the robust performance of the MLM to identify dogs with leptospirosis in a diverse population.

The infecting *Leptospira* serovars vary based on geography and may shift over time.^17^ There are some suggestions that the infecting serovar may also alter the clinical presentation in dogs, including clinicopathologic changes such as platelet count or degree of azotemia.^12^ However, those described studies are based on serology using a limited MAT panel, which does not accurately predict the infecting serovar, so those findings are difficult to confirm. Our study included dogs over 20 y, the training set from the first 18 y, and the test set from the last 2 y of this time period, but all were from a single geographic location and utilized a single clinical laboratory. Indeed, some clinicopathologic differences were noted between these 2 groups of dogs with leptospirosis, yet the MLM was still able to detect leptospirosis with high accuracy. However, future studies should validate this assay in dogs from a wide geographic area through a prospective and multi-institutional study, with larger sample sizes and clinicopathologic data collected from a variety of clinical laboratories with values resulted from different instrumentation, reagent lots, and alternative quality control strategies to determine transferability. Furthermore, all dogs in our study were from a referral hospital, which may not reflect the general population of dogs with leptospirosis. The data may represent more severe clinicopathologic changes because of more advanced disease at the time of referral or alterations as a result of therapy administered before referral. Incorporation of dogs being tested for leptospirosis in a primary care setting would be valuable to determine if MLMs can identify affected dogs with earlier or less severe disease.

MLMs can be implemented through integration into the electronic medical record system, in the clinical diagnostic laboratory record system, or through third-party software applications. Model validation and optimization should be performed for individual clinical laboratories and geographic regions, and it is essential to have post-implementation monitoring of the MLM performance in each population.
